# SnakeBITE: A SNAKEmake-Based Interface for Third-Generation Sequencing Data Analysis

**DOI:** 10.3390/biom16020314

**Published:** 2026-02-16

**Authors:** Andrea Bimbocci, Marta Baragli, Alberto Magi

**Affiliations:** Department of Information Engineering, University of Florence, 50139 Florence, Italy; andrea.bimbocci@unifi.it

**Keywords:** nanopore sequencing, genomics, graphical user interface

## Abstract

In recent years, the use of computational pipelines for the analysis of omic data has become routine in bioinformatics, particularly with the advent of Next-Generation Sequencing (NGS) technologies. These technologies generate vast amounts of data that necessitate sophisticated analysis methods, often requiring programming skills and command-line interface proficiency. This complexity poses challenges for users from various backgrounds, including clinicians and biologists. Current solutions often involve workflow management tools and graphical user interfaces to simplify pipeline creation and execution. However, these tools predominantly cater to NGS data and are not fully adaptable to Third-Generation Sequencing (TGS) data, such as that produced by Oxford Nanopore Technologies (ONT). Here we present SnakeBITE, a modular genomic data analysis pipeline builder based on the Snakemake workflow manager, integrated with an interactive Shiny-based interface. SnakeBITE enables users to configure and execute TGS data analysis workflows locally without requiring programming expertise. The application supports the full ONT genomics data analysis pipeline, including base calling, alignment, variant calling, and annotation. Our results demonstrate SnakeBITE’s capacity to handle various stages of ONT data analysis efficiently, offering a user-friendly and highly customizable tool that bridges the gap between sophisticated data analysis and user accessibility.

## 1. Introduction

Computational pipelines have become a cornerstone of omics data analysis, particularly following the widespread adoption of next-generation sequencing (NGS) technologies, which generate large-scale datasets requiring complex and multi-step computational processing [1]. As sequencing technologies and experimental designs have diversified, the number of professionals involved in data analysis has expanded beyond bioinformatics specialists to include researchers from clinical, biological, and engineering backgrounds. Despite this growing user base, the construction and execution of customized analysis pipelines remain largely dependent on programming expertise and proficiency with command-line interfaces.

Even when addressing similar analytical objectives, multiple computational approaches and tool configurations are often available, and optimal parameterization frequently depends on data-specific characteristics. In practice, the majority of existing solutions require familiarity with scripting languages, environment configuration, and dependency management. Although the adoption of virtual environments (e.g., Conda) and containerization technologies (e.g., Docker and Singularity) has partially alleviated software compatibility issues, these approaches have introduced additional layers of complexity that can hinder accessibility for non-expert users. As a consequence, many clinicians and experimental researchers remain dependent on specialized bioinformatics personnel or external service providers, raising concerns related to scalability, turnaround time, and data confidentiality.

To address these challenges, several workflow management systems and user-oriented platforms have been developed to simplify pipeline creation and execution. Tools such as Snakemake [2], Nextflow [3], and GenPipes [4], along with broader surveys of available solutions [5], have enabled reproducible and scalable automation of NGS analyses. In parallel, graphical and web-based platforms (including Galaxy [6], DolphinNext [7], and iCOMIC [8]) have lowered the barrier to entry by providing user-friendly interfaces and preconfigured or modular workflows. These systems have proven effective across a wide range of NGS applications, either through fully web-based infrastructures or locally deployable solutions. A structured comparison of representative platforms and the corresponding research gaps addressed by *SnakeBITE* is provided in Table 1.

However, the emergence of third-generation sequencing (TGS) technologies has introduced new analytical requirements that are not fully addressed by existing NGS-oriented frameworks. Among these technologies, Oxford Nanopore Technologies (ONT) has gained particular attention due to its ability to generate long reads, support real-time sequencing, and enable novel experimental designs [9,10]. Recent improvements in sequencing chemistry and base-calling algorithms have substantially mitigated earlier limitations related to accuracy [11], further expanding the applicability of ONT data in genomics and other omics disciplines. Nevertheless, the unique characteristics of ONT data necessitate dedicated computational pipelines that differ substantially from those developed for short-read NGS technologies.

Although several user-friendly solutions targeting ONT data analysis are available (such as InterARTIC [12], the Galaxy platform [6], and the EPI2ME suite provided by Oxford Nanopore Technologies [13]) they present notable limitations. InterARTIC is restricted to fixed viral sequencing workflows, EPI2ME offers predefined pipelines with limited customization, and Galaxy does not fully integrate all critical steps of ONT analysis, particularly base calling, due to input size constraints for large-scale datasets.

At present, a comprehensive and easily customizable framework for end-to-end TGS data analysis that is accessible to users from diverse backgrounds remains lacking. To address this gap, we present SnakeBITE, a modular pipeline builder for variant calling based on the Snakemake workflow management system and controlled through an interactive Shiny-based graphical interface [14]. SnakeBITE enables complete ONT genomic data analysis from base calling and alignment to variant detection and functional annotation, while decoupling analytical execution from the need for advanced programming expertise. SnakeBITE is publicly available at https://github.com/Lab-CoMBINE/SnakeBITE (accessed on 16 January 2026).

## 2. Materials and Methods

### 2.1. Software Architecture

SnakeBITE is implemented as a modular and extensible software system composed of four functional components. While the current release focuses on genomic analysis, the underlying architecture is designed to accommodate additional modules, such as transcriptomics and epigenetics, in future updates [15,16]. The system consists of (i) a graphical user interface (GUI) for user interaction and pipeline configuration, (ii) a workflow management layer responsible for pipeline execution, (iii) auxiliary Python scripts mediating communication between the GUI and the workflow engine, and (iv) a collection of third-party bioinformatics tools performing the individual analytical steps.

The strict separation between the GUI and the workflow management layer is inherent to the system design. The workflow engine operates exclusively through command-line interfaces and does not natively provide interactive or graphical components; SnakeBITE bridges this gap by exposing workflow configuration and execution through a web-based interface.

SnakeBITE is designed to operate entirely within local or institutional computing environments. The graphical user interface is deployed as a local web application and is typically accessed via localhost or through secure SSH tunneling when executed on remote servers or high-performance computing systems. No external web services are required for pipeline configuration or execution. All sequencing data, intermediate files, and results remain within the local file system or institutional infrastructure where SnakeBITE is executed. The software does not transmit data externally over the network, nor does it perform any cloud-based processing by default. This execution model ensures full control over data locality and is compatible with data protection requirements in clinical and sensitive research settings.

### 2.2. Graphical User Interface

The GUI is developed using Shiny for Python [14], a reactive web application framework that enables the construction of dynamic user interfaces without direct use of HTML or JavaScript. The interface is composed of pre-defined interactive widgets (e.g., text inputs, sliders, checkboxes, and buttons) whose state is updated in real time in response to user input.

The application logic is divided into a user interface layer, which defines layout and visual components, and a server layer, which implements the reactive behavior, input validation, and generation of configuration files. User selections are translated into structured configuration objects that are subsequently passed to the workflow management system. This design enables real-time feedback, dynamic rendering of interface components, and validation of user-defined parameters prior to workflow execution.

### 2.3. Workflow Management and Execution

Pipeline execution is managed using Snakemake, a Python-based workflow management system designed for reproducible and scalable data analysis. Analytical steps are defined as discrete rules specifying input files, output files, parameters, and executable commands. Dependencies between rules are automatically inferred from input–output relationships, allowing Snakemake to construct a directed acyclic graph (DAG) representing the workflow.

Based on the DAG structure and user-defined computational resources, Snakemake executes rules either sequentially or in parallel. Parallel execution is enabled for independent steps and for analyses sharing common input files, such as variant calling with multiple tools. Execution can be resumed from the last successfully completed step in the event of runtime errors, avoiding redundant recomputation.

Pipeline configuration is specified using human-readable YAML files, generated automatically by the GUI. These configuration files control conditional execution of workflow branches, tool selection, parameter settings, and resource allocation. Execution logs and error messages are recorded to facilitate monitoring and debugging.

### 2.4. Environment Management and Reproducibility

To ensure reproducibility and dependency isolation, SnakeBITE supports execution within virtual environments managed through Conda and container-based solutions where applicable. Each analytical tool can be associated with a dedicated environment to prevent version conflicts and to maintain consistent runtime behavior across different systems.

Custom Python scripts are used to assemble configuration files, validate user input, generate Snakefiles dynamically, and coordinate communication between the GUI and the workflow engine. This modular design improves maintainability and extensibility of the software.

The minimum system requirements of SnakeBITE depend on the third-party tools selected for pipeline execution. As the framework itself acts as an orchestration layer, it does not impose additional computational constraints beyond those required by the underlying software components. Based on the official documentation and typical usage of the integrated tools, a minimum configuration consisting of 8 CPU cores and 16 GB of RAM is sufficient to execute a complete ONT genomic analysis on moderate-size datasets using CPU-based workflows. GPU resources are not mandatory and are only required when GPU-enabled basecalling tools, such as Dorado, are selected. Disk space requirements depend on input data size and intermediate file generation, particularly during basecalling and alignment steps.

### 2.5. External Software and Availability

SnakeBITE integrates a set of established third-party bioinformatics tools covering the main stages of ONT genomic data analysis, including base calling, read alignment, alignment post-processing, variant calling, and variant annotation. All tools are executed externally through the workflow management system and are distributed under their respective licenses. The software packages used in this study and their official sources are listed below.

Basecalling is performed using Dorado (v0.5.0) [17], available from the Oxford Nanopore Technologies GitHub repository (https://github.com/nanoporetech/dorado (accessed on 22 May 2024)), while read alignment is carried out using minimap2 (v2.24) [18], available at https://github.com/lh3/minimap2 (accessed on 22 May 2024). Alignment post-processing, including SAM-to-BAM conversion, sorting, and indexing, is conducted using Samtools (v1.15.1) [19], distributed via https://github.com/samtools/samtools (accessed on 22 May 2024). Structural variant calling is supported through four tools: SVIM (v1.4.2) [20] (https://github.com/eldariont/svim, accessed on 22 May 2024), Sniffles2 (v2.2) [21] (https://github.com/fritzsedlazeck/Sniffles, accessed on 22 May 2024), cuteSV (v1.0.8) [22] (https://github.com/tjiangHIT/cuteSV, accessed on 22 May 2024), and GASOLINE (v1.1) [23] (https://sourceforge.net/projects/gasoline/, accessed on 22 May 2024). These tools operate on aligned BAM files and produce structural variant calls in VCF format. Single-nucleotide variant (SNV) and small insertion/deletion (InDel) calling is performed using Longshot (v0.4.1) [24] (https://github.com/pjedge/longshot, accessed on 22 May 2024), NanoCaller (v3.4.1) [25] (https://github.com/WGLab/NanoCaller, accessed on 22 May 2024), Clair3 (v1.0.4) [26] (https://github.com/HKU-BAL/Clair3, accessed on 22 May 2024), and PEPPER–Margin–DeepVariant (r0.8) [27] (https://github.com/kishwarshafin/pepper, accessed on 22 May 2024). Variant annotation is conducted using AnnotSV (v3.3.6) [28], available at https://github.com/lgmgeo/AnnotSV, (accessed on 22 May 2024), for structural variants, and Variant Effect Predictor (VEP, release 105.0) [29], distributed via the Ensembl repository (https://github.com/Ensembl/ensembl-vep, accessed on 22 May 2024), for SNVs and small InDels. All software tools and their respective versions used for the validation run are summarized in Table 2, and all software packages were obtained from their official repositories or project websites to ensure version traceability and reproducibility.

## 3. Results

### 3.1. SnakeBITE

SnakeBITE is a graphical user interface developed in Shiny for Python that provides an intuitive, widget-based environment for defining ONT genomic analysis pipelines. Through the interface, users can select the desired workflow steps, choose among supported analysis tools, and configure their associated parameters. The application leverages the underlying Snakemake workflow management system to enable automated execution and parallelization of compatible tasks, thereby improving computational efficiency while minimizing the need for manual construction of complex command-line instructions.

The user interface is organized into five navigable pages, accessible from a sidebar menu within the web application. Each page corresponds to a specific stage of pipeline configuration and is designed to guide both inexperienced and expert users through the workflow setup, providing contextual information to ensure correct and reproducible configuration of each analysis step.

### 3.2. The Interface

**Figure 1:**
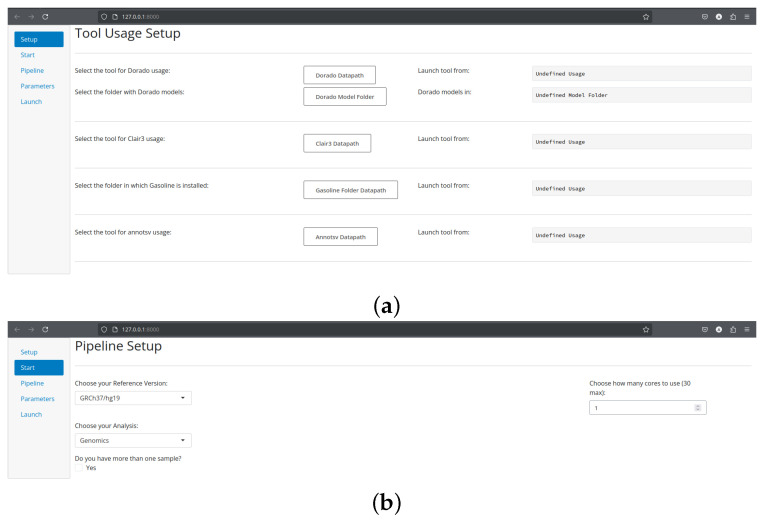
Initial SnakeBITE interface pages for tool setup and global workflow configuration. (**a**) Setup page, where users specify executable paths and environment-related information required to enable the use of pre-installed tools. (**b**) Start page, providing global workflow settings such as reference genome selection, number of CPU cores, and type of omics analysis.

**Figure 2:**
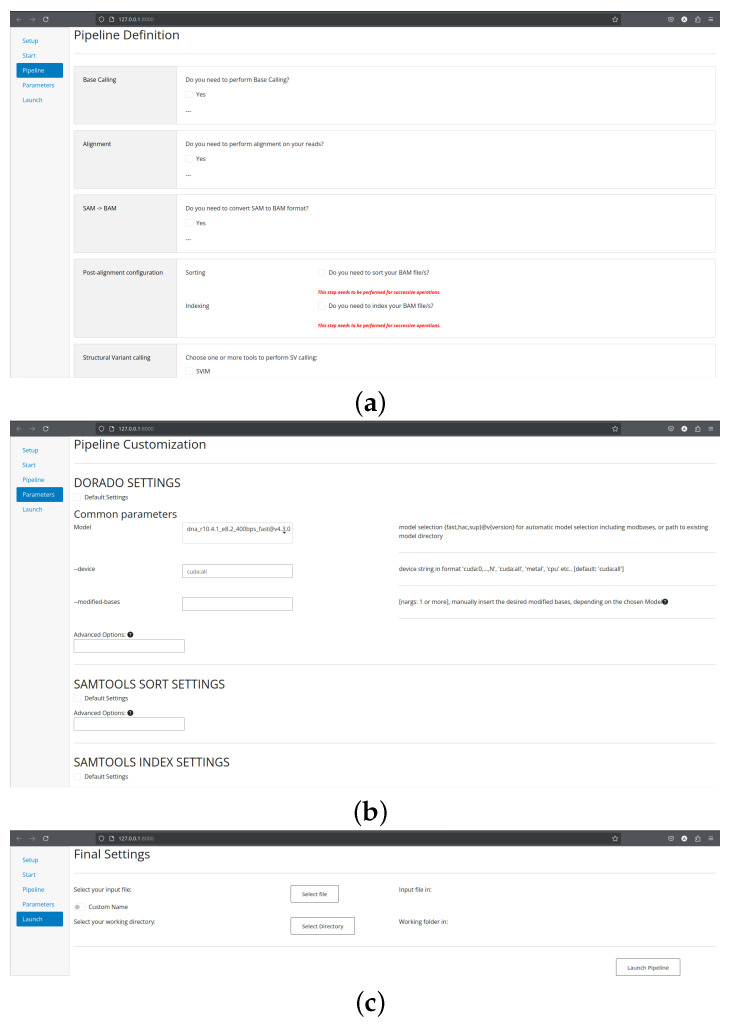
Core SnakeBITE interface pages for workflow definition, parameter customization, and execution. (**a**) Pipeline configuration page, where users select the analysis steps and supported tools for the chosen omics workflow. (**b**) Parameter configuration page, allowing tool-specific parameter customization through guided widgets and contextual descriptions. (**c**) Launch page, where input and output paths are defined and the configured workflow is validated and executed.

1. Setup. The *Setup* page (Figure 1) consists of a one-time configuration form in which users specify initialization parameters for the tools integrated into the SnakeBITE pipeline. When required, executable paths and related environment information must be provided to enable the use of pre-installed software components. This design allows seamless integration of existing analysis tools without requiring redundant installations.
2. Start. The *Start* page (Figure 1) introduces the global settings that define the overall analysis context. These include the type of omics analysis to be performed (genomics, transcriptomics or methylation analysis, with only genomics currently implemented) as well as technical parameters such as the number of CPU cores to allocate and the reference genome to be used. For human genomic analyses, users may select between the widely adopted GRCh37 and GRCh38 assemblies or provide a custom reference genome to accommodate specialized use cases.
3. Pipeline. The *Pipeline* page (Figure 2) enables selection of the analytical steps composing the workflow. Users can activate one or more processing stages and, where applicable, select among multiple supported tools for each step. For analyses with a well-established gold-standard approach, alternative tool choices are intentionally restricted to ensure methodological consistency.
4. Parameters. The *Parameters* page (Figure 2) allows fine-grained customization of the selected analysis steps and tools. Upon selection of any pipeline component, the corresponding tools automatically appear in this section, enabling immediate adjustment of their configuration. Tools requiring no mandatory user-defined parameters expose a single *Default Settings* option, which preserves standard execution behavior. Users may optionally override default configurations by specifying custom parameters through dedicated widgets, accompanied by descriptive flag annotations and direct links to official documentation.
5. Launch. The *Launch* page (Figure 2) collects the final information required to execute the workflow, including input data locations, output directories, and an optional sample identifier. Upon initiation, SnakeBITE dynamically generates a customized Snakefile reflecting the selected configuration and launches the corresponding analysis. Input validation is performed prior to execution, and informative warnings are issued if configuration errors are detected, preventing execution until all issues are resolved.

### 3.3. Complete Pipeline

**Figure 3:**
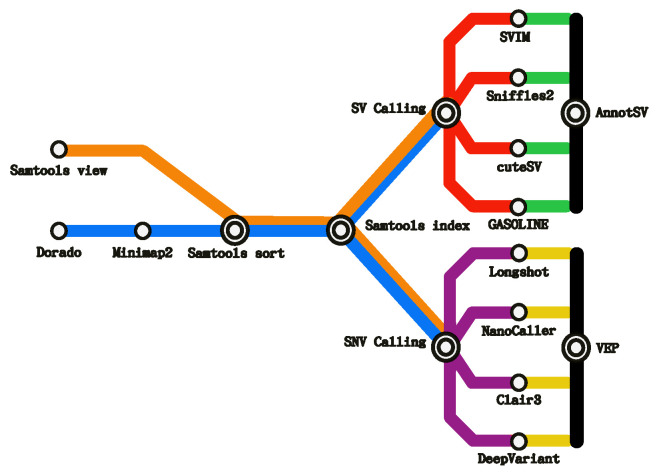
MetroMap-like schematic of the proposed ONT Genomics workflow. The stations represents the tools for each operational step in the data analysis, while each color is linked to a different line of work inside the pipeline. The simple stations represent simple operations, linked to single line of work, while the transfer station are common operations to different lines of work. **Light blue**: pre-variant calling from flowcells operations; **Orange**: pre-variant calling from SAM starting file; **Red**: Structural Variant Calling; **Purple**: Single-Nucleotide Variant Calling; **Green**: Structural Variant annotation; **Yellow**: Single-Nucleotide Variant annotation; **Black**: Annotation operation from VCF input file.

In SnakeBITE, a comprehensive pipeline (Figure 3) for ONT genomic data analysis is proposed. This design choice reflects the rapid growth of third-generation sequencing technologies and the analytical opportunities enabled by long-read data.

Overall, the pipeline is structured into seven logical steps, some of which are inherently sequential, others mutually exclusive, and others amenable to parallel execution. A core design principle of SnakeBITE is modularity: users may execute individual steps independently or assemble a complete workflow in a flexible, block-wise manner, enabling seamless transitions from heterogeneous input formats to a wide range of desired outputs.

1. **Base Calling**. Raw ONT signal data are translated into nucleotide sequences using Dorado, selected in accordance with ONT recommendations for proprietary basecalling software.
2. **Alignment**. Basecalled reads are mapped to a reference genome using Minimap2, enabling standardized downstream analyses. Minimap2 is also directly integrated within Dorado to generate BAM outputs.
3. **SAM-to-BAM Conversion**. Optional conversion of SAM to BAM format is performed using Samtools when uncompressed SAM files are provided as input. Potential conflicts with upstream steps are automatically resolved by the workflow logic.
4. **Sorting and Indexing**. Aligned BAM files are sorted and indexed using Samtools, producing the BAI files required for downstream variant calling. These operations may be executed independently depending on input availability.
5. **Structural Variant Calling**. Structural variants (SVs; ≥50 bp) are identified using one or more of four supported tools: SVIM, Sniffles2, cuteSV, and GASOLINE. These tools were selected based on command-line usability and demonstrated performance in comparative studies [30,31]. When multiple tools are selected, Snakemake enables their parallel execution to reduce computational time.
6. **Single-Nucleotide and InDel Variant Calling**. Small variants (SNVs and InDels) are detected using Longshot, NanoCaller, Clair3, and PEPPER-Margin-DeepVariant. As with SV calling, multiple tools can be executed in parallel, and SNV and SV calling steps may run concurrently when sufficient computational resources are available.
7. **Variant Annotation**. Variant annotation is performed using VEP for SNVs and AnnotSV for SVs, producing annotated VCF or TSV outputs. Annotation can be run as a standalone step from user-provided VCF files or integrated as the final stage of the full workflow, with automatic tool assignment and parallel execution where supported.

SnakeBITE enforces a set of rule-based constraints to prevent incompatible step combinations. At configuration time, the GUI validates user selections and automatically enables/disables steps based on the chosen input type and upstream outputs. At execution time, Snakemake rule dependencies ensure that only the branch consistent with the selected inputs is instantiated (i.e., rules whose required inputs are not produced are not scheduled). When multiple alternatives exist for the same logical operation (e.g., different callers), these are treated as parallel branches operating on the same prerequisite files rather than sequential steps.

### 3.4. Pipeline Validation and Performance Evaluation

After integration of all third-party components, a full test run was performed to validate the correctness, robustness, and computational performance of the SnakeBITE pipeline on ONT genomic data. For reproducibility, publicly available sequencing data generated on an R10.4 flow cell were retrieved from the Oxford Nanopore open-access repository (https://epi2me.nanoporetech.com/giab-2025.01/ accessed on 7 March 2025). The dataset corresponds to the Genome in a Bottle HG002 reference sample (PGP Ashkenazi son, GM24385), sequenced at an average depth of approximately 30×.

The executed workflow covered the complete analytical chain, starting from raw POD5 files and producing annotated VCF outputs. The validation run was configured to include basecalling with integrated alignment using Dorado and minimap2, followed by BAM sorting and indexing with Samtools. Structural variant calling was performed using SVIM, Sniffles2, cuteSV, and GASOLINE, while single-nucleotide variant and small insertion/deletion calling was conducted using Longshot, NanoCaller, Clair3, and PEPPER–Margin–DeepVariant. Variant annotation was carried out using AnnotSV for structural variants and VEP for SNVs and InDels. The SAM-to-BAM conversion step was intentionally excluded, as it is incompatible with the combined basecalling and alignment strategy adopted in this test. All analyses were performed using the GRCh38 reference genome. Unless otherwise required, default parameters were used for all tools. All validation experiments were performed on a Linux-based high-performance computing system equipped with 160 CPU cores, 1.5 TB of RAM, and an NVIDIA A100 GPU with 40 GB of on-board memory. For validation runs, a maximum of 50 CPU cores was allocated, with up to eight concurrent jobs scheduled in parallel by Snakemake. In addition to execution time, overall computational resource usage was considered at the workflow level. As SnakeBITE functions as an orchestration layer built on top of Snakemake, resource consumption is primarily determined by the selected third-party tools rather than by the framework itself, in accordance with the system requirements described in the Materials and Methods section. All variant calling tools were executed using their default parameters and no additional global post-processing or quality filtering was applied to the resulting callsets. Consequently, the reported variant counts include low-confidence calls as defined by each tool’s internal criteria. No explicit masking or filtering of repetitive or low-complexity regions was performed at the workflow level; such regions are handled according to the default behavior of each caller.

Table 3 summarizes the execution times of the initial preprocessing steps. Basecalling and alignment represent the most computationally demanding phase of the pipeline, while sorting and indexing required comparatively negligible execution time.

Structural variant calling performance is reported in Table 4. Execution times varied substantially across SV callers, reflecting differences in algorithmic complexity and parallelization strategies. All tools completed successfully within the same workflow execution, demonstrating effective parallel scheduling and resource allocation.

SNV and small InDel calling results are summarized in Table 5. Considerable differences in runtime were observed among the evaluated tools, with GPU-agnostic methods and single-core tools exhibiting longer execution times. Despite these differences, all variant callers completed within a single coordinated workflow execution.

Finally, annotation performance is reported separately for SVs (Table 6) and SNVs/InDels (Table 7). Annotation runtimes scaled with the size and complexity of the input VCF files and were consistent with the single-core limitation of AnnotSV and the multi-core capabilities of VEP. These results confirm that annotation steps can be efficiently integrated as terminal stages of the workflow without introducing substantial computational overhead.

## 4. Conclusions

The increasing adoption of TGS technologies has led to a growing diversity of professional profiles involved in omics data analysis, spanning bioinformatics, molecular biology, and clinical research. Despite this heterogeneity, the creation and execution of customized analysis pipelines remain largely dependent on command-line expertise and workflow scripting, which can represent a substantial barrier for non-specialist users. In this context, SnakeBITE was developed to address a concrete and persistent need within ONT genomics by providing an accessible, guided, and modular environment for end-to-end data analysis.

The core contribution of SnakeBITE lies in its graphical interface, which abstracts the operational complexity of standard genomic workflows while preserving the flexibility required to compose sequential and parallel analysis steps. Workflow configuration is supported through contextual explanations, tool-specific descriptions, and user-facing warnings that promote informed decision-making and reduce the likelihood of misconfiguration. The deliberate choice of a minimal and uncluttered interface reflects the primary design goal of simplifying the user experience, while the underlying Snakemake workflow has been extensively engineered to dynamically accommodate user-defined configurations generated through the GUI.

The comprehensive test run conducted on publicly available ONT data demonstrated the practical viability of this approach. Execution times varied markedly across pipeline stages and tools, reflecting differences in algorithmic strategies, computational models (CPU- versus GPU-based execution), and parallelization capabilities. Rather than converging toward a single “optimal” configuration, these results highlight the importance of supporting multiple tools for the same analytical task. By enabling users to select one or more tools per pipeline step, SnakeBITE accommodates heterogeneous analytical needs within a single, unified framework.

This flexibility, however, introduces inherent limitations. To ensure consistency and stability, SnakeBITE relies on predefined software versions and fixed default configurations, which may restrict compatibility with newly introduced tool options or non-standard execution modes. While expert users operating directly via command-line interfaces or custom Snakemake workflows can more readily adapt to such changes, SnakeBITE partially mitigates this constraint by allowing advanced users to manually specify additional flags through dedicated interface widgets. Nevertheless, the system remains bounded by the structure of a single, precompiled Snakefile with a configurable parameter space, which may not support unforeseen or atypical tool usages.

Validation was performed on the well-characterized HG002 reference dataset to ensure reproducibility and facilitate comparison with existing benchmarks. The workflow itself is reference-driven and therefore not intrinsically restricted to human data; it can be applied to other species provided an appropriate reference genome and related resources are available. Clinical sample validation was not included in this study due to data access constraints, although the local execution model supports data-sensitive environments.

Despite these constraints, SnakeBITE effectively fulfills its primary objective: enabling users without extensive programming or workflow-management expertise to perform complete ONT genomic analyses in a reproducible and guided manner. By integrating execution logic, resource management, and error handling within an intuitive interface, the software lowers the entry barrier for researchers from clinical and biological backgrounds, reducing reliance on ad hoc scripting or extensive informatics support.

Looking forward, several avenues for future development can further enhance the utility and reach of SnakeBITE. Support for multi-sample parallel execution using shared parameter configurations represents a natural extension of the current framework. Leveraging the modular design described above, the future release of the transcriptomics and methylation profiling modules will significantly broaden the potential user base. Furthermore, the inclusion of pipelines tailored to other sequencing technologies, including PacBio and synthetic long-read platforms, could position SnakeBITE as a versatile solution for laboratories operating across heterogeneous sequencing infrastructures.

From a practical perspective, SnakeBITE addresses several limitations of currently available user-oriented solutions for ONT data analysis. Unlike platforms such as InterARTIC, which are restricted to predefined viral workflows, or EPI2ME, which provides limited customization of analysis steps, SnakeBITE enables users to compose and execute flexible, end-to-end genomic pipelines while retaining full control over tool selection and execution logic. Compared to general-purpose platforms such as Galaxy, which may not seamlessly integrate all stages of ONT analysis due to input size constraints or architectural limitations, SnakeBITE supports complete workflows starting from raw sequencing data and is designed for local or remote execution on institutional computational resources. These characteristics make SnakeBITE particularly suitable for small- to medium-sized laboratories, clinical research units, and sequencing facilities that routinely generate ONT data but lack dedicated bioinformatics personnel. By lowering the technical barrier to pipeline configuration and execution, the software facilitates faster turnaround times, improves reproducibility, and reduces reliance on ad hoc scripting or external service providers. In this context, SnakeBITE can be effectively adopted as an operational tool for routine analyses, pilot studies, and method comparison workflows, bridging the gap between exploratory research and standardized data processing.

Additional improvements may include the integration of data visualization and summary reporting features, such as plots, tables, and interactive graphics, to facilitate downstream interpretation of results. Finally, extending compatibility beyond Unix-based SSH environments to support cloud-based or local execution contexts would address a key accessibility limitation, albeit requiring careful consideration of computational resource management.

In conclusion, SnakeBITE provides a robust and extensible foundation for simplifying third-generation sequencing data analysis. By decoupling informatics expertise from pipeline execution, it offers a practical and user-centered solution that can evolve alongside the rapidly changing landscape of omics technologies.

## Figures and Tables

**Table 1:** Summary of existing ONT-oriented analysis platforms and identified research gaps addressed by SnakeBITE.

Tool/Study	Scope	Main Strengths	Identified Gap
InterARTIC	Viral ONT analysis	User-friendly, automated workflows	Restricted to predefined viral pipelines; limited flexibility
EPI2ME	ONT analysis suite	Integrated official ONT pipelines	Limited customization and parameter control
Galaxy	General NGS platform	Web-based interface, broad tool availability	Does not fully integrate complete ONT workflows; constraints on large datasets
DolphinNext	Workflow platform	GUI-based pipeline composition	Requires pre-defined pipeline structures; not ONT-specific
iCOMIC	Cancer NGS analysis	User-friendly, oncology-focused workflows	Primarily short-read oriented; limited long-read specialization
SnakeBITE	ONT genomic analysis	Modular GUI + Snakemake integration; multi-tool parallel execution	Addresses need for flexible, end-to-end ONT workflows with guided configuration

**Table 2:** Software tools and versions used for the SnakeBITE validation run.

Category	Software	Version
Basecalling	Dorado	0.5.0
Alignment	minimap2	2.24
Post-processing	Samtools	1.15.1
Structural variant calling	SVIM	1.4.2
Structural variant calling	Sniffles2	2.2
Structural variant calling	cuteSV	1.0.8
Structural variant calling	GASOLINE	1.1
SNV/InDel calling	Longshot	0.4.1
SNV/InDel calling	NanoCaller	3.4.1
SNV/InDel calling	Clair3	1.0.4
SNV/InDel calling	PEPPER–Margin–DeepVariant	r0.8
SV annotation	AnnotSV	3.3.6
SNV/InDel annotation	Ensembl VEP	release 105.0
Workflow management	Snakemake	8.10.7
Graphical interface	Shiny for Python	0.6.0
Runtime environment	Python	3.7.16–3.9.12

**Table 3:** Timings of the first operations on ONT data.

Tool	Assigned Cores	Execution Time (d, h, min, s)
Dorado+Minimap2	50	1 d, 7 h, 53 min, 46 s
Samtools sort	50	24 min, 13 s
Samtools index	50	2 min, 21 s

**Table 4:** Timings and number of detected SVs by each variant caller tool.

Tool	Assigned Cores	Execution Time (d, h, min, s)	Number of SVs Detected
SVIM	1	32 min, 16 s	239,889
Sniffles2	8	4 min, 29 s	28,270
cuteSV	8	7 h, 39 min, 27 s	37,342
GASOLINE	8	4 h, 20 min, 56 s	67,402

**Table 5:** Timings and number of detected SNVs by each variant caller tool.

Tool	Assigned Cores	Execution Time (d, h, min, s)	Number of SNVs Detected
Longshot	1	1 d, 19 h, 31 min, 52 s	4,262,990
NanoCaller	8	19 h, 57 min, 21 s	5,189,346
Clair3	8	3 h, 43 min, 50 s	4,479,772
DeepVariant	8	14 h, 1 min, 0 s	5,397,400

**Table 6:** AnnotSV timings for each SV variant caller tool.

Tool	Assigned Cores	SVIM	Sniffles2	cuteSV	GASOLINE
AnnotSV	1	29 min, 50 s	2 min, 2 s	1 min, 57 s	9 min, 11 s

**Table 7:** VEP timings for each SNV variant caller tool.

Tool	Assigned Cores	Longshot	NanoCaller	Clair3	Deepvariant
VEP	8	1 h, 17 min, 57 s	1 h, 29 min, 18 s	1 h, 31 min, 57 s	1 h, 30 min, 43 s

## Data Availability

No new data were created or analyzed in this study.
